# MALDI Imaging Mass Spectrometry Profiling of N-Glycans in Formalin-Fixed Paraffin Embedded Clinical Tissue Blocks and Tissue Microarrays

**DOI:** 10.1371/journal.pone.0106255

**Published:** 2014-09-03

**Authors:** Thomas W. Powers, Benjamin A. Neely, Yuan Shao, Huiyuan Tang, Dean A. Troyer, Anand S. Mehta, Brian B. Haab, Richard R. Drake

**Affiliations:** 1 Department of Cell and Molecular Pharmacology and Experimental Therapeutics and MUSC Proteomics Center, Medical University of South Carolina, Charleston, South Carolina, United States of America; 2 Hollings Cancer Center Biorepository and Tissue Analysis Resource, Medical University of South Carolina, Charleston, South Carolina, United States of America; 3 Departments of Pathology and Microbiology and Molecular Cell Biology, Eastern Virginia Medical School, Norfolk, Virginia, United States of America; 4 Drexel University College of Medicine, Department of Microbiology and Immunology and Drexel Institute for Biotechnology and Virology, Doylestown, Pennsylvania, United States of America; 5 Laboratory of Cancer Immunodiagnostics, Van Andel Research Institute, Grand Rapids, Michigan, United States of America; University of Nebraska Medical Center, United States of America

## Abstract

A recently developed matrix-assisted laser desorption/ionization imaging mass spectrometry (MALDI-IMS) method to spatially profile the location and distribution of multiple N-linked glycan species in frozen tissues has been extended and improved for the direct analysis of glycans in clinically derived formalin-fixed paraffin-embedded (FFPE) tissues. Formalin-fixed tissues from normal mouse kidney, human pancreatic and prostate cancers, and a human hepatocellular carcinoma tissue microarray were processed by antigen retrieval followed by on-tissue digestion with peptide N-glycosidase F. The released N-glycans were detected by MALDI-IMS analysis, and the structural composition of a subset of glycans could be verified directly by on-tissue collision-induced fragmentation. Other structural assignments were confirmed by off-tissue permethylation analysis combined with multiple database comparisons. Imaging of mouse kidney tissue sections demonstrates specific tissue distributions of major cellular N-linked glycoforms in the cortex and medulla. Differential tissue distribution of N-linked glycoforms was also observed in the other tissue types. The efficacy of using MALDI-IMS glycan profiling to distinguish tumor from non-tumor tissues in a tumor microarray format is also demonstrated. This MALDI-IMS workflow has the potential to be applied to any FFPE tissue block or tissue microarray to enable higher throughput analysis of the global changes in N-glycosylation associated with cancers.

## Introduction

Tissues obtained from surgeries or diagnostic procedures are most commonly preserved in formalin-fixed paraffin-embedded (FFPE) tissue blocks. These tissues are fixed in formalin and processed as paraffin-embedded tissue blocks. The embedding process preserves the cellular morphology and allows tissues to be stored at room temperature, causing FFPE fixation to be used by many tissue banks and biorepositories [1], [2]. For cancer biomarker discovery, FFPE tissues are particularly attractive because they are archived for years and are much more widely available than cryopreserved tissue. When combined with clinical outcomes, FFPE tissues are a rich source of samples for biomarker discovery and validation in retrospective studies. While the fixation method has many benefits, the formalin treatment results in the formation of methylene bridges between the amino acids of the proteins, complicating further analysis by mass spectrometry. There has been continued progress in improving extraction methods of trypsin digested peptides from FFPE tissues in recent years, in parallel with improved high resolution sequencing analysis of peptides by mass spectrometry [3], [4]. Incorporation of multiple FFPE tumor tissue cores in a tissue microarray (TMA) format also has proven to be effective for immunohistochemistry analysis of potential biomarker candidates [5], and TMAs are increasingly being used for validation of alterations in protein expression associated with emerging genetic mutation phenotypes and transcriptional profiling studies [6], [7]. The main advantages of experiments performed with TMAs are the ability to include multiple cores from the same subject tumors, improved sample throughput, statistical relevance and multiplexed analysis of diverse molecular targets [5], [8]. Thus, it is possible to place up to 100 samples with duplicates and controls on a single slide. When correlated with associated clinical outcomes, this provides a powerful method for biomarker discovery and validation while minimizing reagent use and assuring that each core in the TMA is treated under identical conditions.

It is well documented that malignant transformation and cancer progression result in fundamental changes in the glycosylation patterns of cell surface and secreted glycoproteins [9]–[11]. Glycosylation of proteins are post-translational modifications most commonly involving either N-linked addition to asparagine residues or O-linked additions to serine or threonine residues. Current approaches to evaluate glycosylation changes generally involve bulk extraction of glycans and glycoproteins from tumor tissues for analysis by mass spectrometry or antibody array platforms, however, this disrupts tissue architecture and distribution of the analytes. Broad affinity carbohydrate binding lectins and a small number of glycan antigen antibodies can be used to target glycan structural classes in tissues, but not individual glycan species. Additionally, these detection methods for global alterations in glycosylation requires staining on many adjacent tissue sections, making large scale assessments on many samples difficult, expensive and time consuming. There are only a few reported studies examining glycosylation related changes of proteins or glycolipids in FFPE cancer tissues [12]–[14], and these focus primarily on determining the levels of the protein carriers or glycosyltransferases through immunostaining.

One potential approach to assess glycan changes in tissues is matrix-assisted laser desorption/ionization imaging mass spectrometry (MALDI-IMS). This technique has been used to directly profile multiple protein [15], [16], lipid [17], [18] and drug metabolite [19]–[21] in tissue, generating molecular maps of the relative abundance and spatial distribution of individual analytes linked to tissue histopathology. MALDI-IMS analysis of peptides following trypsin digestion of FFPE TMAs have also been reported [22]–[24]. Recently, our group reported a MALDI-IMS method workflow to directly profile N-linked glycan species in fresh/frozen tissues [25]. Adapting this method for the analysis of N-glycans in FFPE tissues would serve to extend the application of the technique to larger retrospective sample sets and TMAs.

In this report, we describe the application of MALDI-IMS glycan imaging to various formalin-fixed tissues. Formalin-fixed mouse kidney tissues were used to optimize antigen retrieval, PNGaseF digestion and glycan detection conditions for MALDI-IMS. This was followed by N-glycan analysis of clinical FFPE tissue blocks from prostate and pancreatic cancers, as well as a commercial tissue microarray of hepatocellular carcinoma (HCC). Glycan identity was confirmed by on-tissue collision-induced dissociation (CID) and off-tissue permethylation analysis. An optimized MALDI-IMS workflow is presented that allows routine simultaneous analysis of 30 or more glycans per FFPE tissue, including TMA formats. The approach is amenable to any FFPE tissue, and represents an additional molecular correlate assay for use with the TMA format. Furthermore, depending on the construction of the TMA and targeted tumor type, the approach has the potential to identify novel glycan biomarker panels for cancer detection and prognosis. To our knowledge, this represents the first instance of using MALDI-IMS to profile N-glycans in FFPE tissue blocks or TMAs.

## Materials and Methods

### Materials

The glycan standard NA2 was obtained from ProZyme (Hayward, CA). Trifluoroacetic acid, sodium hydroxide, dimethyl sulfoxide, iodomethane and α-cyano-4-hydroxycinnamic acid (CHCA) were obtained from Sigma-Aldrich (St. Louis, MO). HPLC grade methanol, ethanol, acetonitrile, xylene and water were obtained from Fisher Scientific (Pittsburgh, PA). ITO slides were purchased from Bruker Daltonics (Billerica, MA) and Tissue Tack microscope slides were purchased from Polysciences, Inc (Warrington, PA). Citraconic anhydride for antigen retrieval was from Thermo Scientific (Bellefonte, PA). Recombinant Peptide N-Glycosidase F (PNGaseF) from *Flavobacterium meningosepticum* was expressed and purified as previously described [25].

### FFPE Tissues and TMA

All human tissues used were de-identified and determined to be not human research classifications by the respective Institutional Review Boards at MUSC and Van Andel. Mouse kidneys were excised from euthanized C57BL/6 mice and immediately placed in 10% formalin prior to processing for routine histology and paraffin embedding. Mice were housed in an Institutional Animal Care and Use Committee-approved small animal facility at MUSC, and tissues obtained were harvested as part of approved projects unrelated to glycan tissue imaging. A liver TMA was purchased from BioChain consisting of 16 cases of liver cancer in duplicates, and one adjacent non-tumor tissue for each case. Tissues were from 14 male and two female patients with an average age of 47.5 with a range of 33 to 68 years old, with additional information provided in Table S1. A de-identified prostate tumor FFPE block, stored for 10 years representing a Gleason grade 6 (3+3)/stage T2c adenocarcinoma from a 62 year old Caucasian male, was obtained from the Hollings Cancer Center Biorepository at the Medical University of South Carolina. A pathologist confirmed the presence of approximately 10% prostate cancer gland content in the sample. A de-identified large-cell undifferentiated pancreatic carcinoma FFPE tissue section with low CA19-9 staining was obtained from the Van Andel Institute Biospecimen Repository. For each section analyzed, histological analysis and staining with hematoxylin and eosin (H & E) were performed.

### Washes for Deparaffinization and Rehydration

Tissue and TMA blocks were sectioned at 5 µm and mounted on positively charged glass slides measuring 25×75 mm, compatible with the Bruker slide adaptor plate. The slides were heated at 60°C for 1 hr. After cooling, tissue sections were deparaffinized by washing twice in xylene (3 minutes each). Tissue sections were then rehydrated by submerging the slide twice in 100% ethanol (1 minute each), once in 95% ethanol (one minute), once in 70% ethanol (one minute), and twice in water (3 minutes each). Following the wash, the slide was transferred to a coplin jar containing the citraconic anhydride buffer for antigen retrieval and the jar was placed in a vegetable steamer for 25 minutes. Citraconic anhydride (Thermo) buffer was prepared by adding 25 µL citraconic anhydride in 50 mL water, and adjusted to pH 3 with HCl. After allowing the buffer to cool, the buffer was exchanged with water five times by pouring out ½ of the buffer and replacing with water, prior to replacing completely with water on the last time. The slide was then desiccated prior to enzymatic digestion. Tris buffer pH 9–10 was also effective, but citraconic anhydride buffer was used for all experiments in this study.

### N-glycan MALDI-IMS

An ImagePrep spray station (Bruker Daltonics) was used to coat the slide with a 0.2 ml aqueous solution of PNGaseF (20 µg total/slide) as previously described [25]. Adjacent control tissue slices lacking PNGaseF were generated by covering them with a glass slide during the spraying process. Following application of PNGaseF, slides were incubated at 37°C for 2 hr in a humidified chamber, then dried in a desiccator prior to matrix application. α-Cyano-4-hydroxycinnamic acid matrix (0.021 g CHCA in 3 ml 50% acetonitrile/50% water and 12 µL 25%TFA) was applied using the ImagePrep sprayer. Released glycan ions were detected using a Solarix dual source 7T FTICR mass spectrometer (Bruker Daltonics) (m/z = 690–5000 m/z) with a SmartBeam II laser operating at 1000 Hz, a laser spot size of 25 µm. Images of differentially expressed glycans were generated to view the expression pattern of each analyte of interest using FlexImaging 4.0 software (Bruker Daltonics). Following MS analysis, data was loaded into FlexImaging Software focusing on the range m/z = 1000–4000 and reduced to 0.95 ICR Reduction Noise Threshold. Observed glycans were searched against the glycan database provided by the Consortium for Functional Glycomics (www.functionalglycomics.org). Glycan structures were generated by Glycoworkbench [26] and represent putative structures determined by combinations of accurate m/z, CID fragmentation patterns and glycan database structures.

### Permethylation of Tissue Extracted N-glycans

PNGaseF sprayed mouse kidney tissue slides were incubated for 2 hr at 37°C; 50 µL water was applied on top of the tissue and incubated for 20 minutes to extract the released native N-glycans. The water was removed from the tissue, and then concentrated under vacuum by centrifugation. Permethylation was performed as described [25], and glycans analyzed by MALDI. Masses detected in the permethylation experiments were searched against the permethylated glycan database provided by the Consortium for Functional Glycomics (www.functionalglycomics.org).

### Collision-Induced Dissociation of N-linked Glycans

Glycan standards were spotted on a stainless steel MALDI plate using CHCA matrix and desiccated to yield a homogenous layer. Tissues were prepared as previously described for MALDI imaging of FFPE tissues. 10 spectra of 1000 laser shots with a laser frequency of 1000 Hz were averaged for each spectra provided. The collision energy varied between 60–70V.

### TMA Statistics

Mass spectra from TMA tissue Regions of Interest (ROIs) representing each tissue core were exported directly from FlexImaging and analyzed using an in-house workflow. The peak lists were first deconvoluted followed by calculating the mean peak intensity of points in each ROI, resulting in a monoisotopic peak list corresponding to signal intensity in each region. Comparison of tumor versus non-tumor was accomplished with a Wilcoxon rank sum test. Individual peaks were also evaluated to discriminate between tumor and non-tumor using receiver operator characteristic curves.

## Results

### Analysis of Formalin-Fixed Mouse Kidneys and Human Cancer Tissues

**Figure 1:**
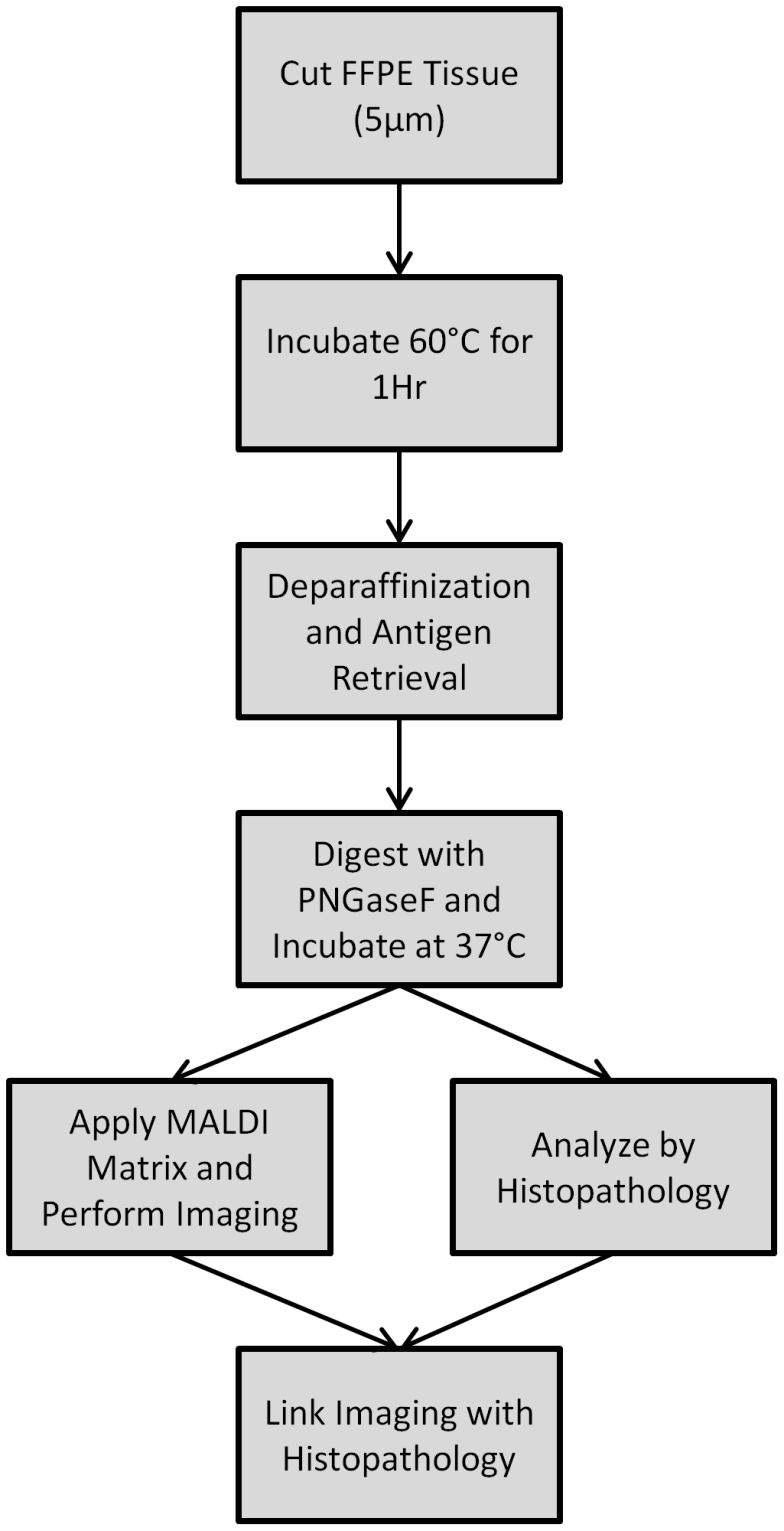
Schematic of the methodology for imaging N-glycans from FFPE tissues. Prior to enzyme application, FFPE blocks are cut at 5 µm, incubated, deparaffinized and undergo antigen retrieval. PNGaseF is then applied and the slide is incubated before MALDI-IMS. The data is then linked with histopathology either on the same tissue slice or a serial tissue slice.

**Figure 2:**
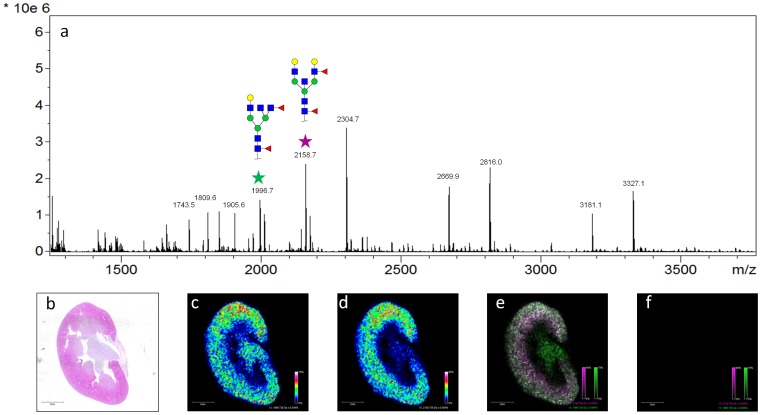
MALDI-IMS of N-Glycans on Mouse Kidney Tissue. Two mouse kidneys were sliced at 5 um prior to proceeding with the MALDI-IMS workflow. One tissue was covered with a glass slide during PNGaseF application to serve as an undigested control tissue. An average annotated spectra from the tissue that received PNGaseF application is provided (a). Tissue regions were assessed by H&E stain (b). The labeled peaks correspond to native N-glycans that have been reported for the mouse kidney on the Consortium for Functional Glycomics mouse kidney database. Two of these ions were selected and their tissue localization was assessed. Hex4dHex2HexNAc5 at m/z = 1996.7 (c) is located in the cortex and medulla while Hex5dHex2HexNAc5 m/z = 2158.7 (d) is more abundant in the cortex of the mouse kidney. An overlay image of these two masses is also shown (e), as well as the corresponding image from untreated PNGaseF control tissues (f).

Mouse kidney tissues were fixed in formalin and used as an initial model system to develop MALDI-IMS glycan imaging workflows for FFPE tissues. These tissues were chosen due to the availability of reference glycan structures and spectra (Consortium for Functional Glycomics; www.functionalglycomics.org), and previous MALDI-IMS glycan imaging data from our laboratory for fresh/frozen tissue analysis [21]. A summary workflow schematic is provided (Figure 1). Tissues were cut at 5 microns, deparaffinized and rehydrated in sequential xylene/ethanol/water rinses, followed by antigen retrieval in citraconic anhydride pH 3. The rehydrated tissues were sprayed with PNGaseF, incubated for glycan release, sprayed with CHCA matrix, and then analyzed by MALDI-IMS. While all data shown herein uses CHCA, 2,5-dihydroxybenzoic acid (DHB) matrix could also be used successfully for N-glycan imaging of FFPE tissues. As shown in Figure 2, there were multiple ions detectable only in the tissue incubated with PNGaseF that were not present in the control tissue with no PNGaseF application. Different glycans were distributed across the cortex or medulla regions. For example, a Hex4dHex2HexNAc5 ion (m/z = 1996.74) is present in the cortex and medulla (Figure 2c), while a Hex5dHex2HexNAc5 glycan (m/z = 2158.76) is more specific to the cortex (Figure 2d). An overlay of the MALDI-IMS images for these two ions from the PNGaseF treated sections (Figure 2e) and the control tissue (Figure 2f) demonstrates that these two ions are released by PNGaseF. In control kidney tissues that were only sprayed with aqueous PNGaseF solution lacking enzyme, or tissue slices that were not processed by antigen retrieval plus and minus PNGaseF digestion, only matrix ions or paraffin/formalin polymer were detected (data not shown). A summary glycan image panel of 28 glycan ions detected in these kidneys, sodium adducts and observed/expected m/z values is provided in Figure S1. Additionally, N-glycans were extracted from the tissue following on-tissue PNGaseF digestion, permethylated and analyzed by MALDI. A representative spectra from this analysis is provided in Figure S2. These permethylated values were also compared with MALDI reference spectra for mouse kidney glycans from the Consortium for Functional Glycomics. The imaged glycan ions were correlated to the reference spectra glycans, illustrated in Figure S3, and could be matched to all 28 glycan species highlighted in the reference spectra.

**Figure 3:**
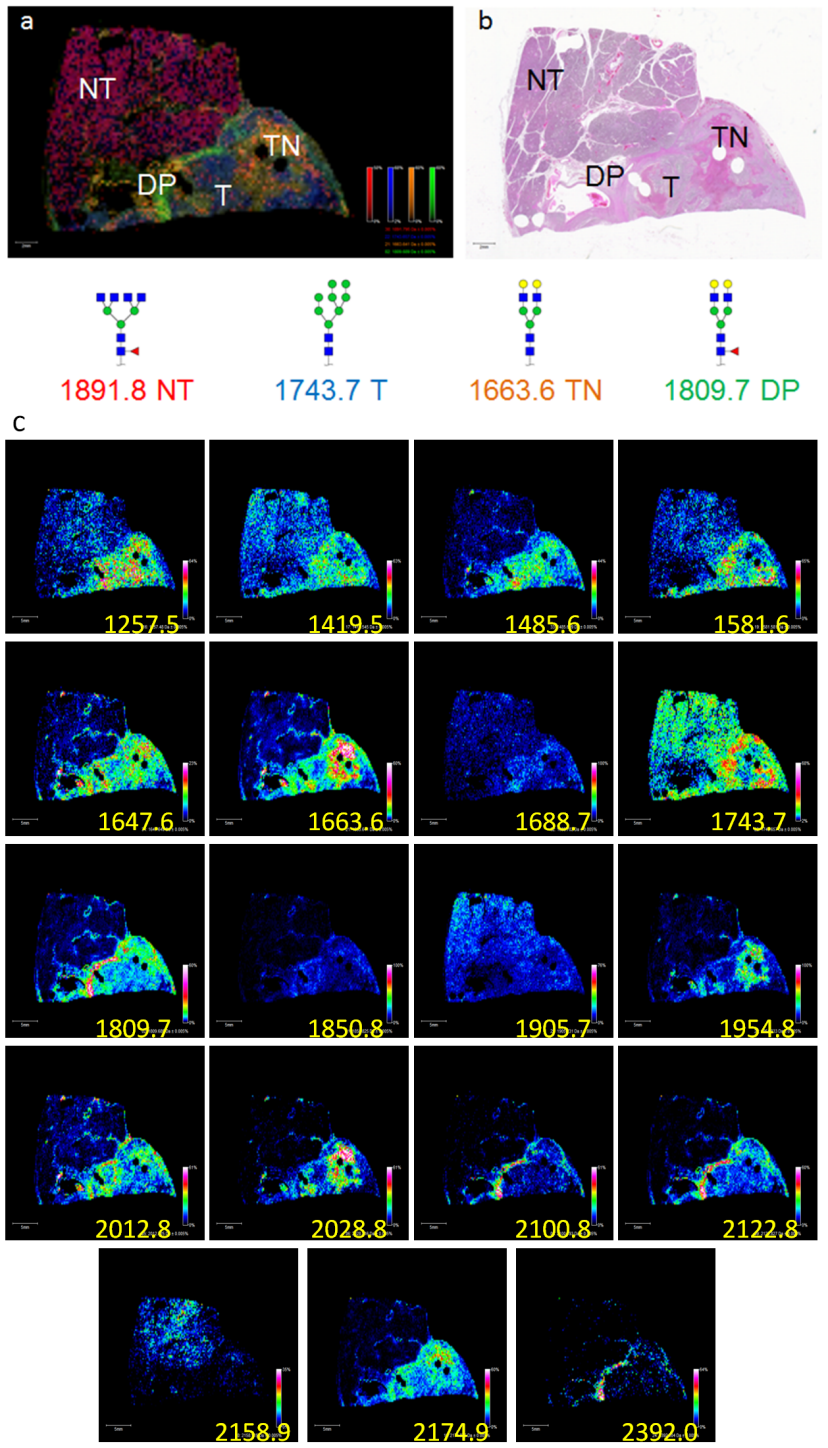
MALDI-IMS of a Human Pancreas FFPE Tissue Block. An FFPE block of pancreatic tissue from a human patient was cut at 5 um prior to and selected for MALDI-IMS. Histopathology found four unique regions in the H&E of this tissue block. The tissue block contained tumor tissue, non-tumor tissue, fibroconnective tissue representing desmoplasia surrounding the tumor tissue, and necrotic tissue (b). MALDI-IMS was able to distinguish these four regions based off of specific ions after MALDI-IMS. M/z = 1891.80 (red) is found in the non-tumor (NT) region of the pancreas and corresponds to Hex3dHex1HexNAc6, while m/z = 1743.64 (blue) represents Hex8HexNAc2 and is predominant in the tumor region (T) of the tissue. Desmoplasia (DP) is represented by m/z = 1809.69 (green) corresponding to Hex5dHex1HexNAc4. In the region where necrosis was identified (TN), m/z = 1663.64 (orange) was elevated corresponding to Hex5HexNAc4. Image spectra were acquired at 200 µm raster. (c). Representative individual glycan images for the pancreatic FFPE tissue slice.

We next assessed whether the method was compatible with two representative archived pathology FFPE tissue blocks, one for pancreatic cancer and one for prostate cancer. A section of human pancreatic cancer tissue of complex histology was processed, incubated with PNGaseF and glycans detected by MALDI-IMS (Figure 3). Different N-glycans were detected that could distinguish between non-tumor, tumor, tumor necrotic and fibroconnective tissue regions. A representative glycan image overlay of four m/z values that correspond to the sodium adducts of potential N-glycan species is shown in Figure 3a, each representing a specific region of the tissue (Figure 3b). A glycan of m/z = 1891.80 (red)/Hex3dHex1HexNAc6 was detected primarily in the non-tumor region of the pancreas, while a glycan of m/z = 1743.64 (blue)/Hex8HexNAc2 was predominant in the tumor region of the tissue. A region of desmoplasia surrounding the tumor region, an area of increased extracellular matrix proteins and myofibroblast-like cells resulting in a dense fibrous connective tissue [27], is represented by a glycan of m/z = 1809.69 (green)/Hex5dHex1HexNAc4. A region of tumor necrosis is represented by a different glycan of m/z = 1663.64 (orange)/Hex5HexNAc4. Additional examples of tissue distributions of other individual glycan species are shown in Figure 3c.

**Figure 4:**
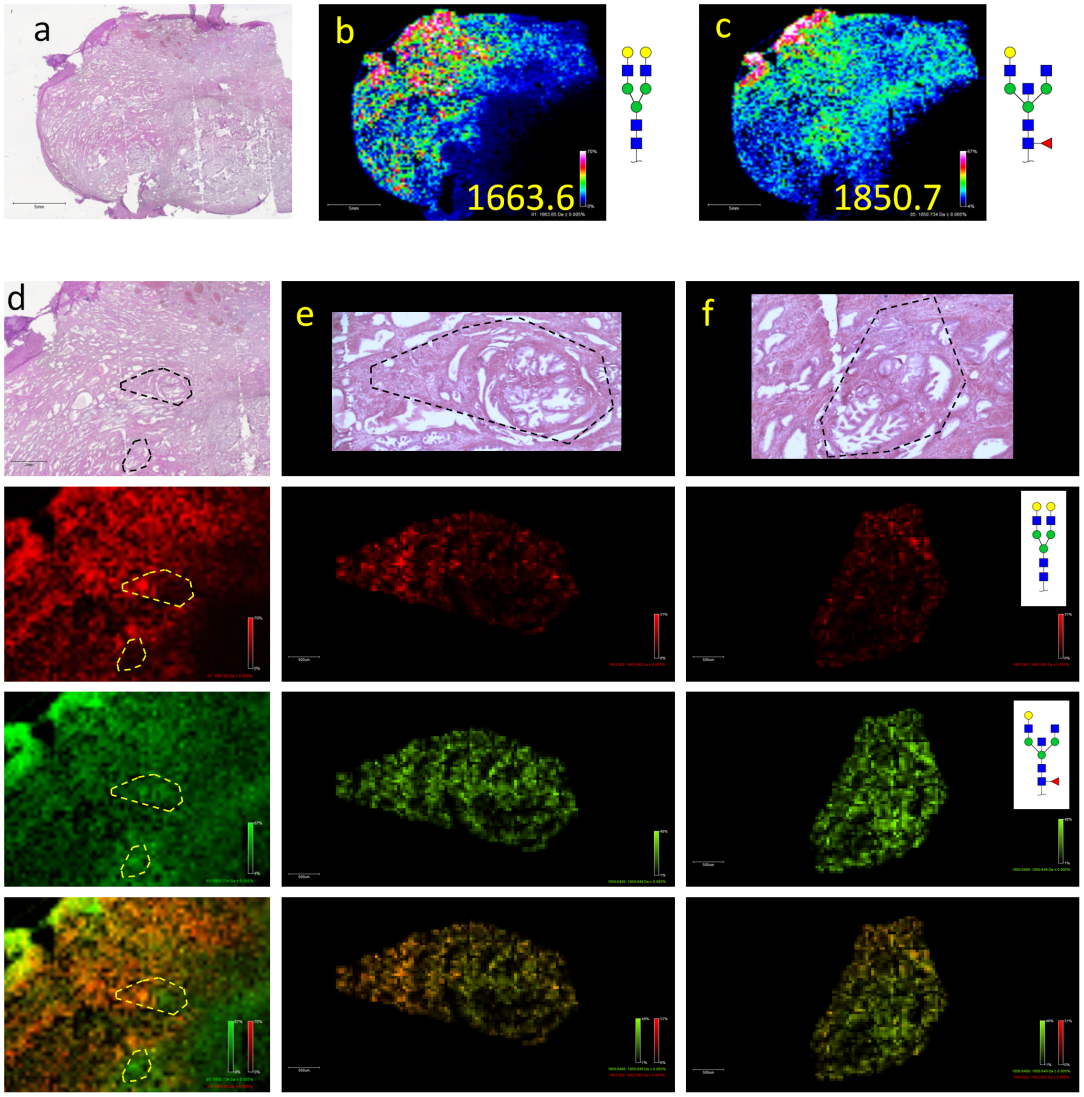
MALDI-IMS of a Human Prostate FFPE Tissue Block. An archived FFPE block of prostate tissue from a human patient was cut at 5 µm and prepared for MALDI-IMS glycan analysis, (a). H&E image. A global glycan imaging experiment performed with a raster of 225 µm demonstrated a heterogeneous expression of two glycan ions (b). at m/z = 1663.56 and (c). m/z = 1850.65. Stromal versus gland distribution were further assessed in a high resolution experiment at 50 µm raster (d–f). Column (d) indicates a 2× amplification of the H&E, and distribution of the same two glycans are shown at this magnification for m/z = 1663.56 (red) and m/z = 1850.65 (green), and an overlay image. Column (e) (enlargement of upper region shown in d). and (f) (enlargement of lower region shown in d), show two highlighted regions of stroma and glands enhanced at 10× resolution, with the same colors and glycans shown for column (d).

A human prostate tissue block containing both tumor and non-tumor gland regions was also analyzed by MALDI-IMS. A heterogeneous N-glycan distribution reflective of the tissue histology was observed, and as an example of stroma and gland distributions, two glycan ions and two sub-regions within the tissue are highlighted in Figure 4. Distribution of glycans of m/z = 1663.56 (Hex5HexNAc4) and m/z = 1850.65 (Hex4dHex1HexNAc5) are shown in Figure 4b and 4c. A higher resolution tissue imaging analysis was done for selected regions as marked in the panel, with the H&E images (Figure 4d–4f) highlighting stroma and gland substructures. In both instances, m/z = 1850.65 is present in both the stroma and glands, while m/z = 1663.56 is predominantly located in the stroma. An overlay of these two ions depicts the stroma as an orange color, demonstrating the presence of both red and green, while the glands are predominantly green. The distribution of other representative individual glycan ions is provided in Figure S4, including the distribution of high-mannose glycan species (Man5–Man9) associated with the heterogeneous tumor region in this tissue.

### On-tissue Glycan Fragmentation and Structural Composition

**Figure 5:**
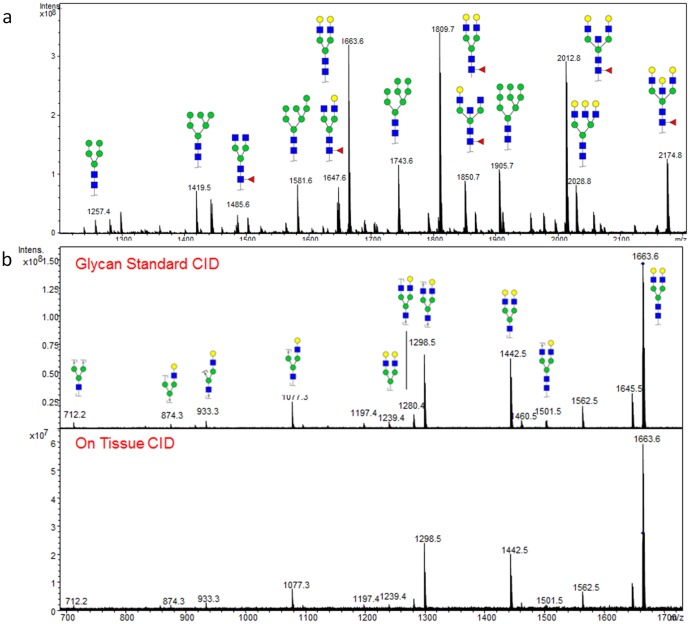
Comparison of the Fragmentation Pattern of a Glycan Standard with the same Ion on Tissue. (a). A representative MALDI spectra for native N-linked glycans from pancreatic cancer FFPE tissue. (b). NA2 glycan standard (m/z = 1663.6) was fragmented using CID, revealing a variety of cleavages across glycosidic bonds as demonstrated in the spectrum (a). When the same ion was fragmented on the pancreatic tissue, the fragmentation pattern was the same, verifying that we were detecting Hex5HexNAc4 in the human pancreas.

The glycan structures identified by imaging of the FFPE tissue blocks were assigned based on the comparison to permethylated species, glycan reference databases and previous studies [24]. An on-tissue approach to further verify N-glycan structures was done using collision-induced dissociation (CID) directly on the human pancreatic tissue. Released native glycans from pancreatic cancer FFPE tissues were used as a source for on-tissue CID analysis, and a representative MALDI spectra of these glycans is shown in Figure 5a. For comparison, a Hex5HexNAc4 (m/z = 1663.6) purified standard (also termed NA2) was spotted on a stainless steel MALDI target plate and fragmented by CID, generating a robust fragmentation pattern of glycans for this ion as previously reported by Harvey et al [28]. The same glycan ion was abundant in pancreatic tissue after PNGaseF release of N-glycans (Figure 3) and was selected for CID. As shown in Figure 5b, the CID fragmentation pattern of m/z 1663.6 in pancreatic tissue was the same as the N-glycan standard, confirming detection of NA2 directly in pancreatic tissue (Figure 3). Mass shifts due to loss of individual sugar ions were detected, such as Hex (resulting in m/z = 1502.5), HexNAc (resulting in m/z 1460.5), and Hex + HexNAc (resulting in m/z = 1298.5) (Figure 5b). An ion at m/z = 712.2, which has been previously characterized [28] as the sodium adduct of Hex3HexNAc1, was also detected. The structures of 13 other glycan ions were confirmed using this CID approach, and additional fragmentation data and spectra are provided in Figures S5 & S6.

### Glycan MALDI-IMS of a Hepatocellular Carcinoma Tissue Microarray

**Figure 6:**
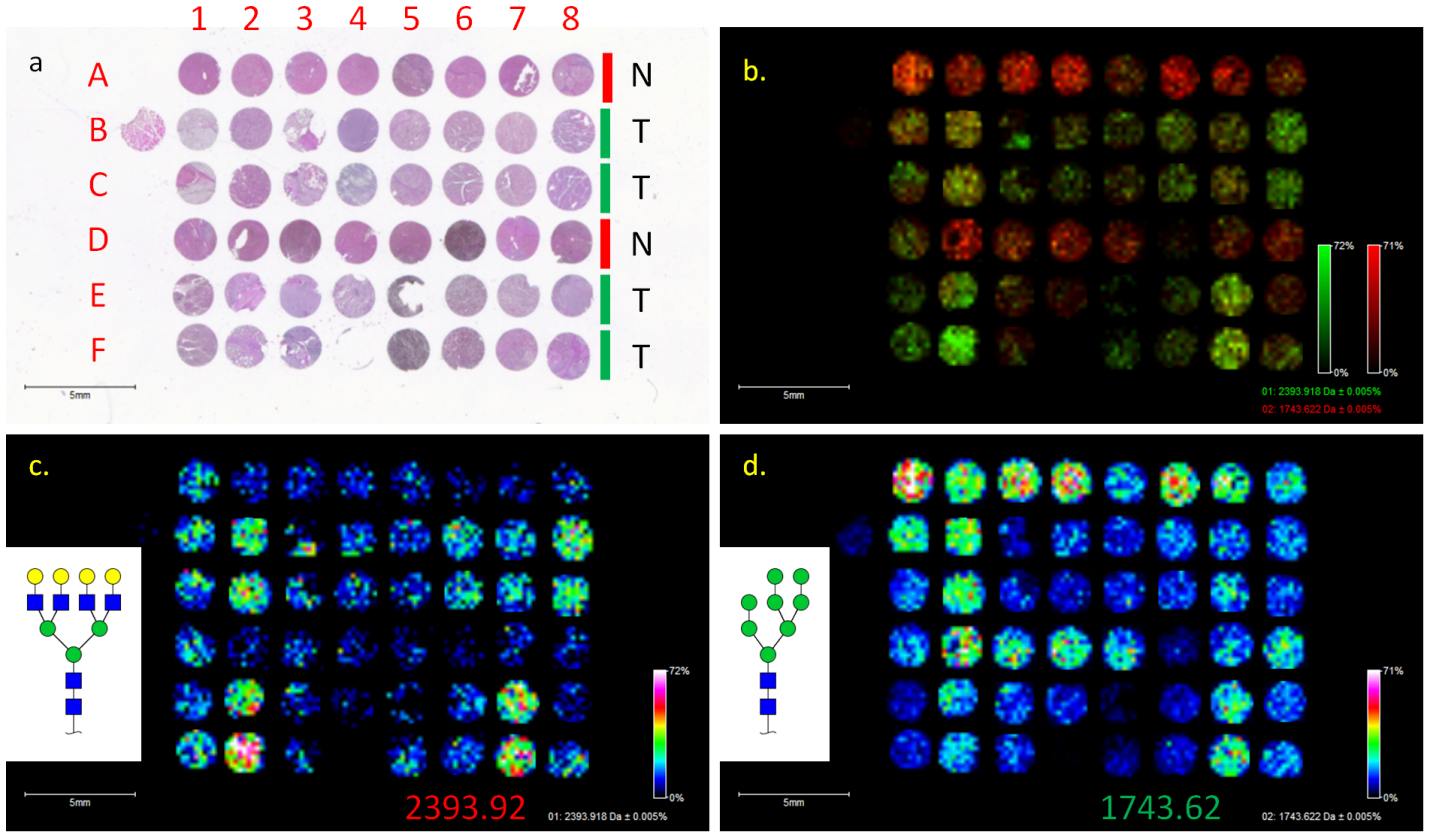
N-Glycan Imaging of a Liver TMA. A liver TMA purchased by BioChain consisting of 2 tumor tissue cores and one normal tissue core from 16 patients was imaged (200 µm raster). The H&E (a) provides the TMA location (red letters and numbers) and classifies whether the row is tumor (green bar) or non-tumor (red bar). M/z = 2393.95 (c) and m/z 1743.64 (d) were able to distinguish between hepatocellular carcinoma and uninvolved liver tissue. An overlay of these ion demonstrates that m/z = 2393.95 is elevated in tumor tissue and m/z = 1743.64 is elevated in normal tissue (b). Statistical data for these two ions is provided in Table 1.

The ability to perform N-glycan analysis on FFPE tissues potentially enables the analysis of multiple FFPE tissue cores in a TMA format. Initial experiments were performed using a commercially available hepatocellular carcinoma (HCC) TMA (BioChain) consisting of samples from 16 individual patients, with two tumor tissue cores and one non-tumor tissue core per patient (Figure 6). Additional patient data are provided in Table S1. Glycan MALDI-IMS was done as described for the other FFPE tissues, and imaging data for two representative glycan ions at m/z = 2393.92 (Hex7HexNAc6) and m/z = 1743.62 (Hex8HexNAc2) are shown in Figure 6. Analysis of the cumulative MALDI spectra and detected ions for each tissue core were processed and compared using an in-house bioinformatic workflow followed by statistical analysis. Of the 176 identified ions from the HCC TMA, 132 were increased in tumor cores, and 83 ions had a p-value<0.05. Interestingly 78 (94%) of the significantly different ions were elevated in tumor cores. After cross-referencing this list of 176 ions with glycans presented in this paper and our previous study [21], 26 N-glycans of high-confidence structure determinations were selected, listed in Table 1. Of these 26 known glycans, ion intensities of 13 species were significantly different in tumor and normal tissue (p<0.05), and 21 were increased in tumor relative to normal. FlexImaging was then used to demonstrate the distribution and relative ion intensities of each glycan across the TMA (images provided in Figure S7). Additionally, ROC curves were used to evaluate how well each of the glycan ion intensities discriminates tumor versus non-tumor. Of the 176 identified ions, 61 had area under the ROC curve (AuROC)>0.80, indicating they are strong classifiers. For two glycans at m/z = 2393.95 (Hex7HexNAc6) and m/z = 1743.64 (Hex8HexNAc2), both had an AuROC>0.80 and a p-value<0.05, with m/z 2393.95 being elevated in tumor tissue and m/z 1743.64 being elevated in non-tumor tissue, as demonstrated by the log2-fold change value (tumor/non-tumor) (Figure 6). In the overlay (Figure 6b) tumor tissue is predominantly green and non-tumor tissue is predominantly red, confirming results from our statistical analysis. This data, although from limited numbers of samples, demonstrates the potential ability of a panel of glycans to be used to accurately discriminate cell types or outcomes on a TMA by MALDI-IMS.

**Table 1:** Comparison of Tumor and Non-Tumor Glycans Detected in Hepatocellular Carcinoma Tissue Microarrays.

m/z	AuROC	log2-fold change	p-val
1866.76	0.895	3.045	<.001
2393.95	0.879	1.819	<.001
2378.01	0.869	3.495	<.001
1743.64	0.831	−0.967	<.001
1850.78	0.827	1.953	<.001
1257.42	0.821	0.780	<.001
1501.60	0.813	2.223	0.001
2686.02	0.764	4.316	0.003
1905.71	0.742	−0.572	0.007
1298.44	0.742	0.910	0.007
2012.82	0.734	1.165	0.010
2320.89	0.707	2.146	0.022
2540.03	0.707	1.493	0.022
3271.15	0.657	2.004	0.082
2028.74	0.657	0.360	0.082
1647.62	0.649	0.683	0.099
1581.57	0.635	−0.190	0.135
2174.89	0.633	0.598	0.141
1976.71	0.617	0.398	0.197
2100.77	0.597	0.295	0.266
1954.79	0.593	−0.114	0.307
1419.48	0.577	−0.076	0.400
1809.67	0.548	0.973	0.598
1485.54	0.488	0.149	0.902
1663.66	0.490	0.255	0.920
1282.46	0.494	0.703	0.991

A list of 26 monoisotopic ions that were identified in the HCC TMA were cross-referenced against a library of known glycan m/z values listed in this paper or our previous paper (21). The ability of individual glycan ions to distinguish tumor tissue from non-tumor tissue were assessed using AuROC, log2-fold changes (tumor/non-tumor), and p-values. Localization of m/z = 2393.95 and m/z = 1743.62 (highlighted in yellow) are depicted in Figure 6.

## Discussion

Multiple N-linked glycans can be directly profiled from FFPE tissue blocks and TMAs while maintaining intact architecture. The basic methodology, which mirrors that of MALDI-IMS analysis of peptides in FFPE tissues and TMAs [18]–[20], requires deparaffinization and antigen retrieval prior to PNGaseF application. The ability to adapt the N-glycan imaging method originally designed for fresh/frozen tissues [25] to encompass FFPE tissue and TMA blocks increases the scope and speed of glycan-based studies that can be performed in tissues. In initial studies of formalin-fixed mouse kidney slices, the MALDI-IMS workflow successfully identified all 28 of the glycans in the mouse kidney database provided by the Consortium for Functional Glycomics. Many of the structures of these glycans were verified by permethylation (Figure S2) and CID experiments (Figures S5 & S6). As observed with the mouse brain [25], these glycans were not homogenously present across the entire mouse kidney slice, but were either predominantly located in the cortex, or distributed across the cortex and medulla (Figure S1). This unique distribution of N-glycans associated with tissue sub-structure or disease status was also observed in human pancreas and prostate tissue slices. In the pancreas, an overlay of four different glycans was able to map the normal pancreas tissue, tumor pancreas tissue, a region of desmoplasia, and a necrotic region (Figure 3a). Similarly, an overlay of two glycans could distinguish between prostate stroma and glands (Figure 4).

In general, the peak intensities of PNGaseF-released glycans in the FFPE tissues seems to be more intense than that obtained with fresh/frozen tissue sections. This may be a result of the more extensive heating and washing steps required in the deparaffinization and rehydration steps. It is this increased detection sensitivity that facilitated CID fragmentation of N-glycans directly from the tissue (Figure 5b, Figures S5 & S6). Under the conditions used, CID generated mainly fragments across the glycosidic bonds, which were useful in characterizing that the structure was an intact hexose or HexNAc. This did not provide any information regarding anomeric linkages between sugar residues. The amount of fragmentation observed was directly related to the relative intensity of each parent N-glycan ion, and inversely related to the mass of the parent ion observed. This is typified by the extensive fragmentation of two glycans of m/z = 1663.50 and m/z = 1809.64 (Figure 5b, Figures S5 & S6).

One drawback to using FFPE tissues is residual polymer from the paraffin block adjacent to the tissue. Detection of this polymer is more predominant in the lower mass range of the imaging runs, and can overlap with potential glycan masses, complicating detection and further statistical analysis. This polymer can be observed in the average spectra of the mouse kidney tissue after PNGase application (Figure 2a) from m/z = 1250–1300, 1450–1500, and 1650–1700. An additional key to distinguishing polymer peaks is the analysis of spectra from the non-PNGaseF treated control tissues. Particularly for the TMA format, the ion selection program that we report can detect and account for polymer peaks relative to glycan ions. These polymer peaks seem to vary in terms of intensity compared to N-glycan ions depending on what tissue is being used. It is possible that this variation is a function of different formalin formulations, variations is tissue processing (i.e. amount of time in formalin), storage time or variations in the tissue itself [1], [2]. These considerations will be further monitored and evaluated as more glycans from FFPE tissues are analyzed.

In relation to potential cancer diagnostic applications, the most significant aspect to developing a method to image N-glycans on FFPE tissue blocks could be the ability to use TMAs for high-throughput glycan-based experiments. Not only does the method increase the number of tumor samples that can be analyzed in one experiment, but it could also be used to compare the glycans detected in a TMA core versus the larger source FFPE tissue. N-glycan MALDI-IMS of the HCC TMA (Figure 6) is provided as an example, but we have already obtained initial glycan profiling data from TMAs representing prostate, kidney, lung, breast, colon and pancreatic cancers. In the HCC TMA, a statistically significant increase in tetra-antennary N-glycan (m/z = 2393.95) and decrease in Man-8 glycan (m/z = 1743.64) was detected in HCC cores compared to adjacent non-tumor tissue (Figure 6 and Table 1). The tetra-antennary N-glycan has been previously demonstrated to be elevated in HCC compared to matched adjacent non-tumor tissue by Mehta et al. [29]. Continued investigations will be performed on whether these two ions can distinguish between matched HCC and non-tumor tissues in other HCC TMAs. Our data analysis identified a total of 176 ions in the tissue, with the majority of significantly different ions being increased in HCC relative to non-tumor tissue, including 21 known or previously identified glycans. It is unclear how this trend of increased glycan levels relates specifically to tumor related biochemical changes, though the role of glycosylation in tumor development is well documented. Future work will also focus on determining the identity of the remaining ions to distinguish other glycan species from the aforementioned polymer peak contaminants.

Currently, MALDI-IMS provides a new approach to effectively visualize and evaluate N-glycan localization in tissue sections. It does not solve the known limitations of MALDI analysis of underivatized glycans like loss of sialic acids, nor does it provide anomeric linkage information for N-glycan structure. Established tandem mass spectrometry methods of glycan extraction, modification and fragmentation are more capable of providing this structural information. Combining the glycan tissue maps generated by MALDI-IMS to target regions of interest for further tandem mass spectrometry analysis of glycans could be a new synergistic approach to more effectively identify tumor-associated glycans and glycoproteins in situ. Use of other glycosidases like sialidase, as we have previously reported [25], or fucosidases, could further extend the utility of the combined methods. Overall, the ability to effectively profile N-glycans on FFPE tissue blocks and TMAs provides new opportunities to evaluate glycan profiles associated with disease status.

## Supporting Information

Figure S1 **Panel of Mouse Kidney N-Glycans.** Ions detected in the kidney with enzyme application were compared to the control tissue. Ions that were only observed in the tissue following PNGaseF application were compared to the glycans found in the mouse kidney database on the Consortium for Functional Glycomics. The panel provides the glycan species, the projected mass for the sodium adduct, and our observed mass for the sodium adduct.
(TIF)

Figure S2 **Permethylation of Mouse Kidney N-Glycans.** Mouse kidney N-glycans were extracted from the imaging slide after PNGaseF application and digestion. Glycans were dried down and underwent permethylation as previously described. The permethylated m/z values were then compared to the permethylation data from the Consortium for Functional Glycomics mouse kidney database (www.functionalglycomics.org).
(TIF)

Figure S3 **Panel of Mouse Kidney N-Glycans Linked to Known Glycan Database.**
(TIF)

Figure S4 **Individual N-Glycans from Prostate Cancer FFPE Tissue.** The orange ovals highlight the areas of heterogeneous tumor for high mannose glycans.
(TIF)

Figure S5 **CID of N-Glycans from Human Pancreas Tissue I.**
(TIF)

Figure S6 **CID of N-Glycans from Human Pancreas Tissue II.**
(TIF)

Figure S7 **Images From Ions Corresponding to N-Glycans.** The Ions identified in Table 1 were viewed in FlexImaging Software.
(TIF)

Table S1 **Patient Data Summary for Hepatocellular Carcinoma TMA from Biochain.**
(TIF)
