# Supplementary figures and images for: MALDI Imaging Mass Spectrometry Profiling of N-Glycans in Formalin-Fixed Paraffin Embedded Clinical Tissue Blocks and Tissue Microarrays
Source: PLoS One. 2014 Sep 3;9(9):e106255. doi: 10.1371/journal.pone.0106255 (PMC4153616; doi:10.1371/journal.pone.0106255)

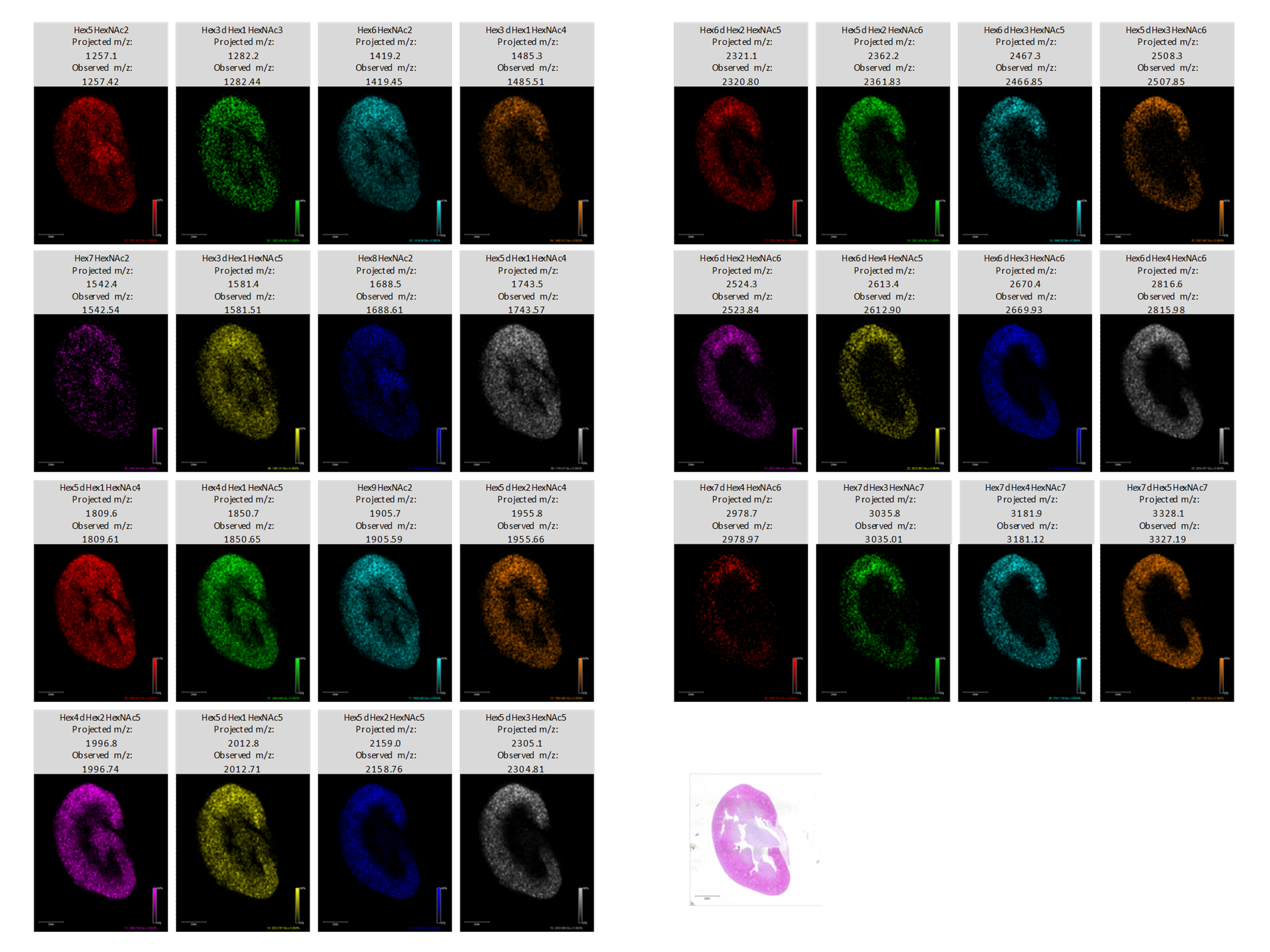

Supplement: Figure S1 — Panel of Mouse Kidney N-Glycans. Ions detected in the kidney with enzyme application were compared to the control tissue. Ions that were only observed in the tissue following PNGaseF application were compared to the glycans found in the mouse kidney database on the Consortium for Functional Glycomics. The panel provides the glycan species, the projected mass for the sodium adduct, and our observed mass for the sodium adduct. (TIF) [file pone.0106255.s001.tif]

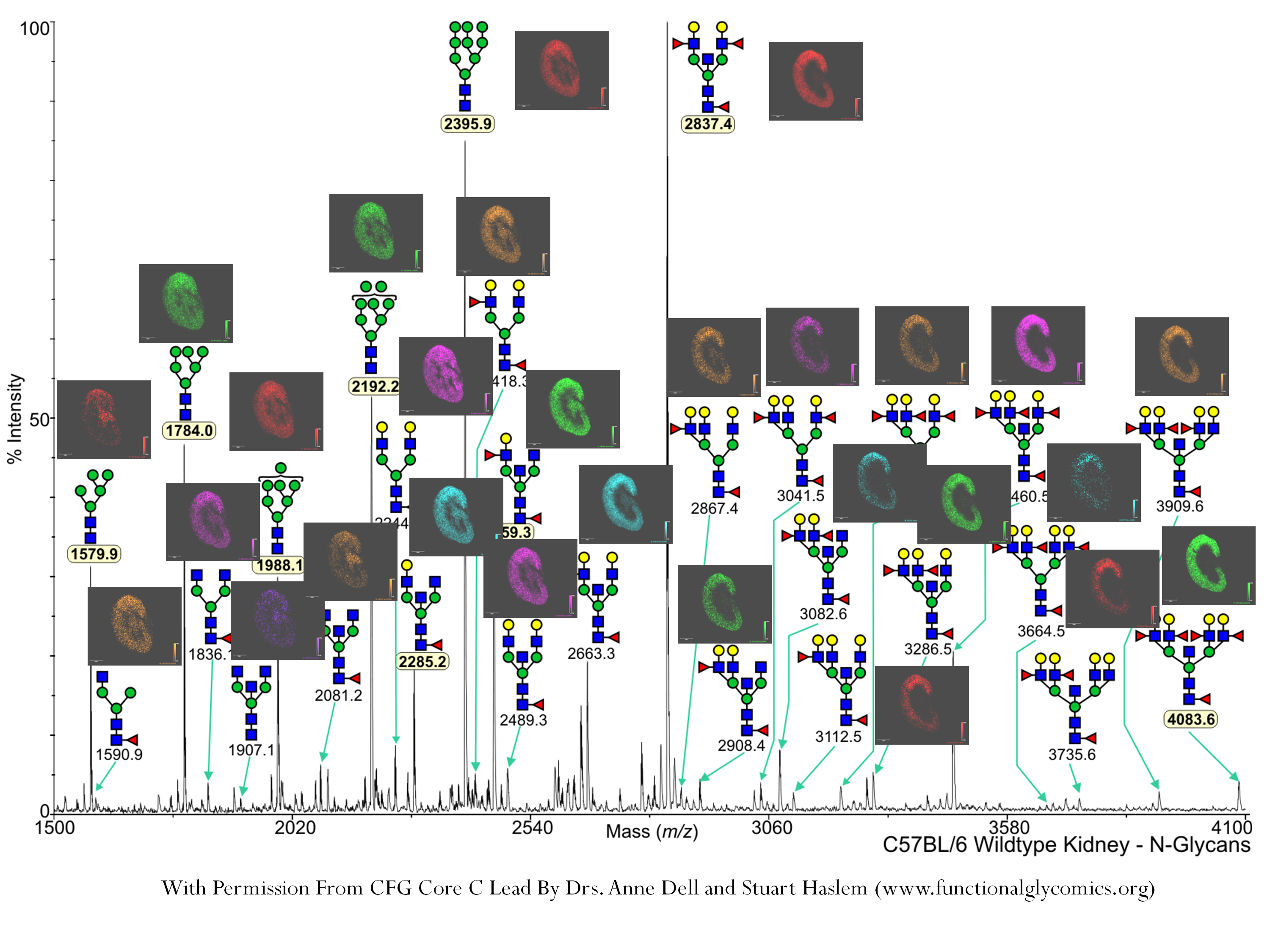

Supplement: Figure S3 — Panel of Mouse Kidney N-Glycans Linked to Known Glycan Database. (TIF) [file pone.0106255.s003.tif]

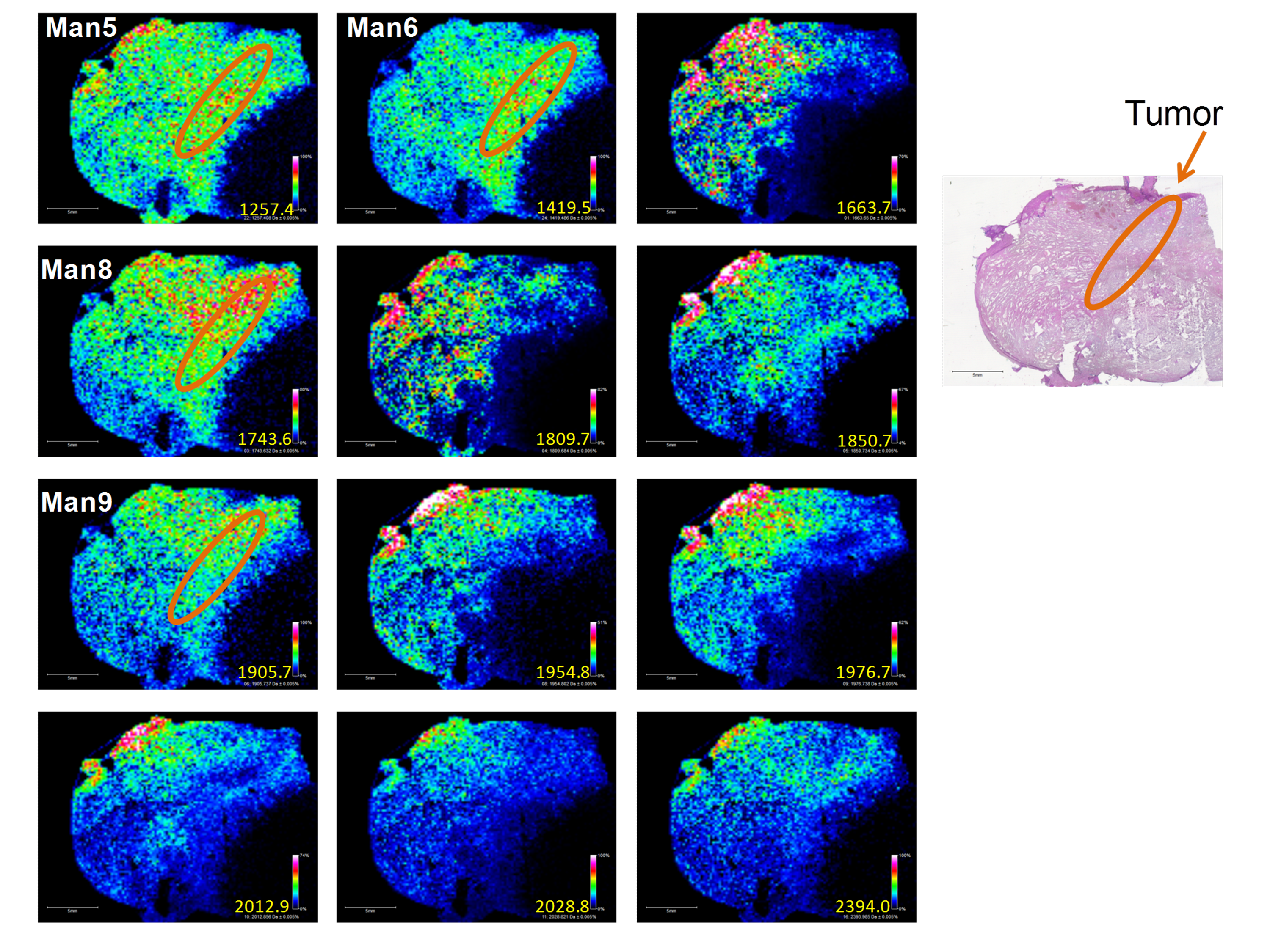

Supplement: Figure S4 — Individual N-Glycans from Prostate Cancer FFPE Tissue. The orange ovals highlight the areas of heterogeneous tumor for high mannose glycans. (TIF) [file pone.0106255.s004.tif]

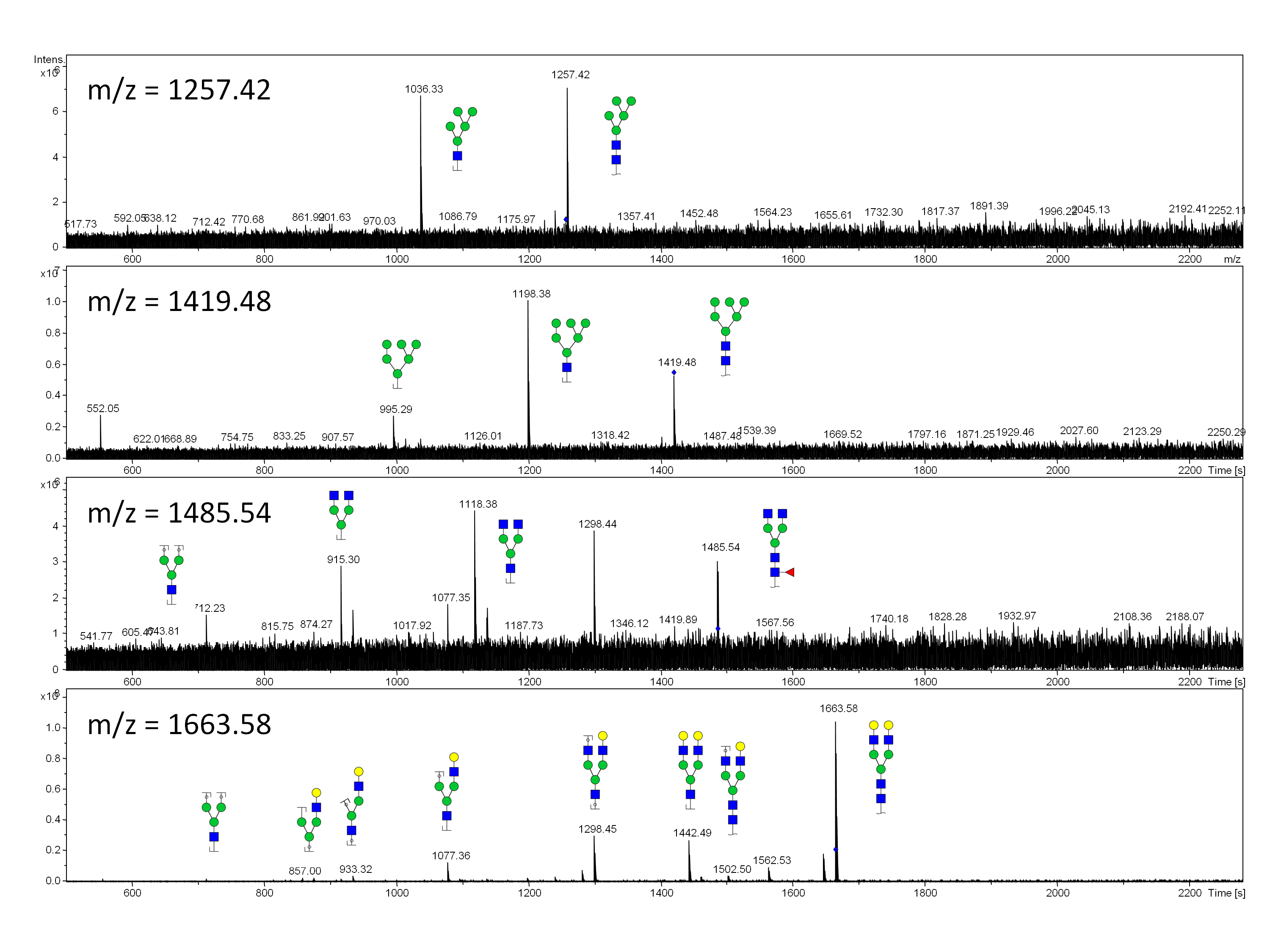

Supplement: Figure S5 — CID of N-Glycans from Human Pancreas Tissue I. (TIF) [file pone.0106255.s005.tif]

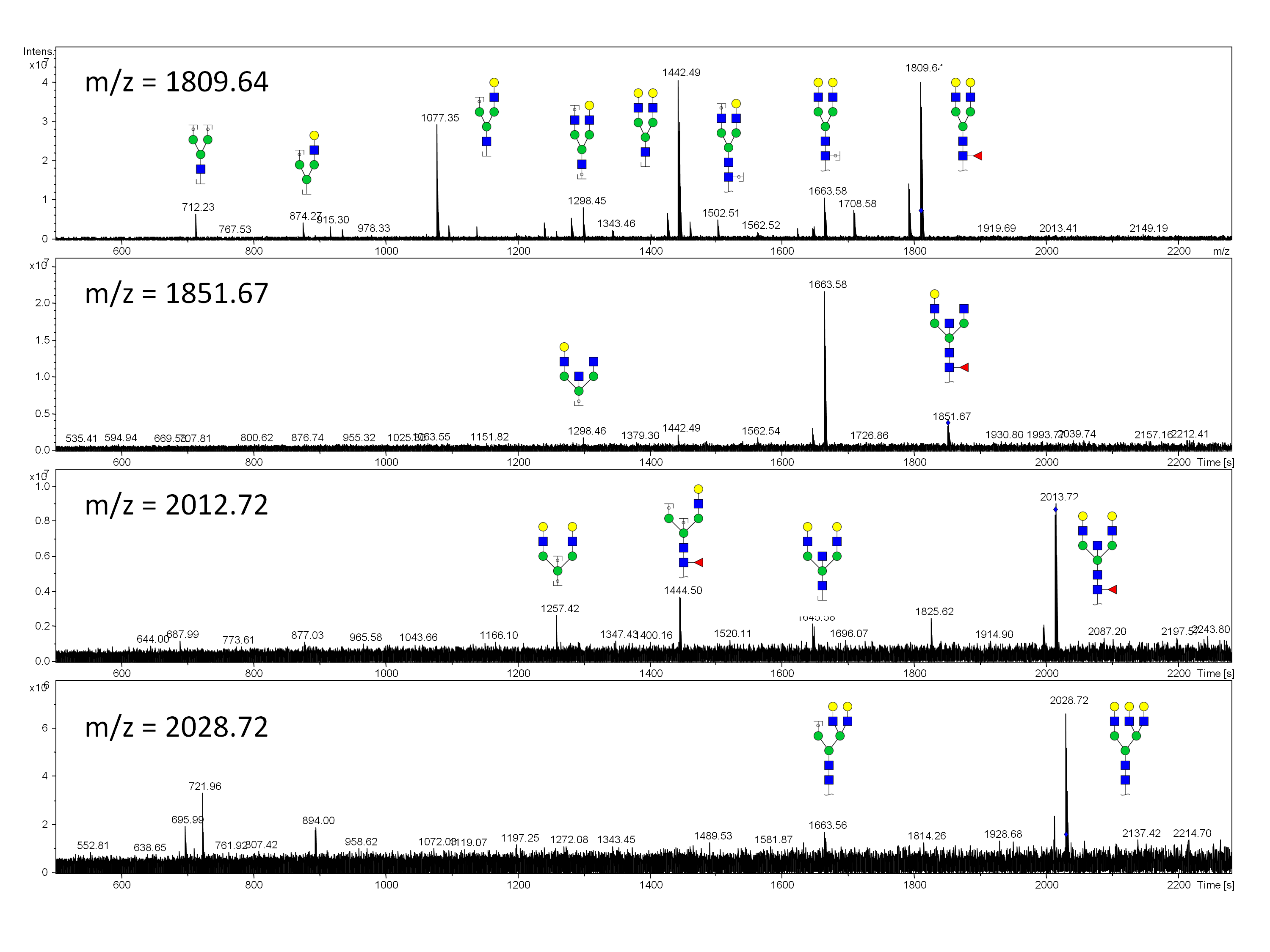

Supplement: Figure S6 — CID of N-Glycans from Human Pancreas Tissue II. (TIF) [file pone.0106255.s006.tif]

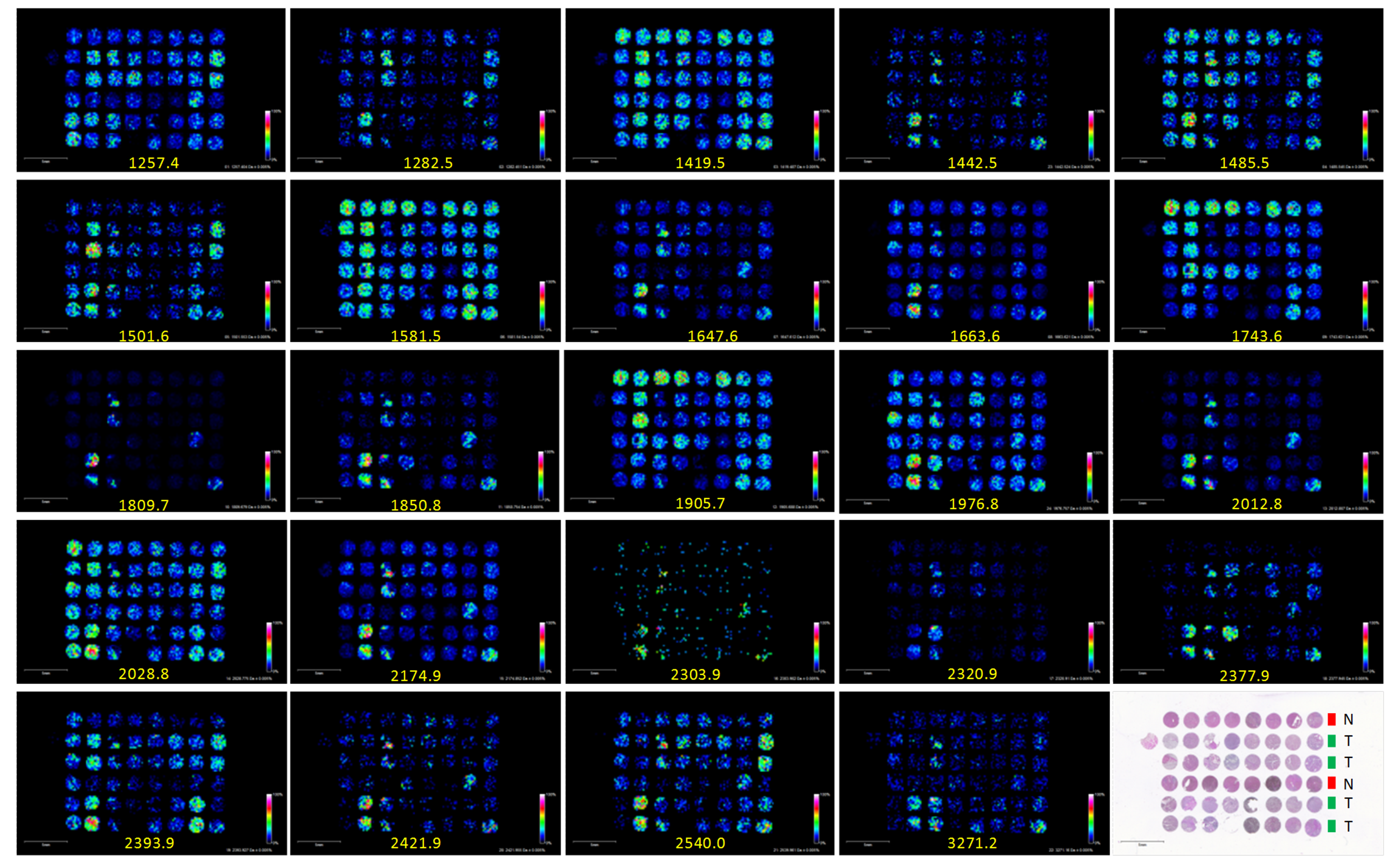

Supplement: Figure S7 — Images From Ions Corresponding to N-Glycans. The Ions identified in Table 1 were viewed in FlexImaging Software. (TIF) [file pone.0106255.s007.tif]

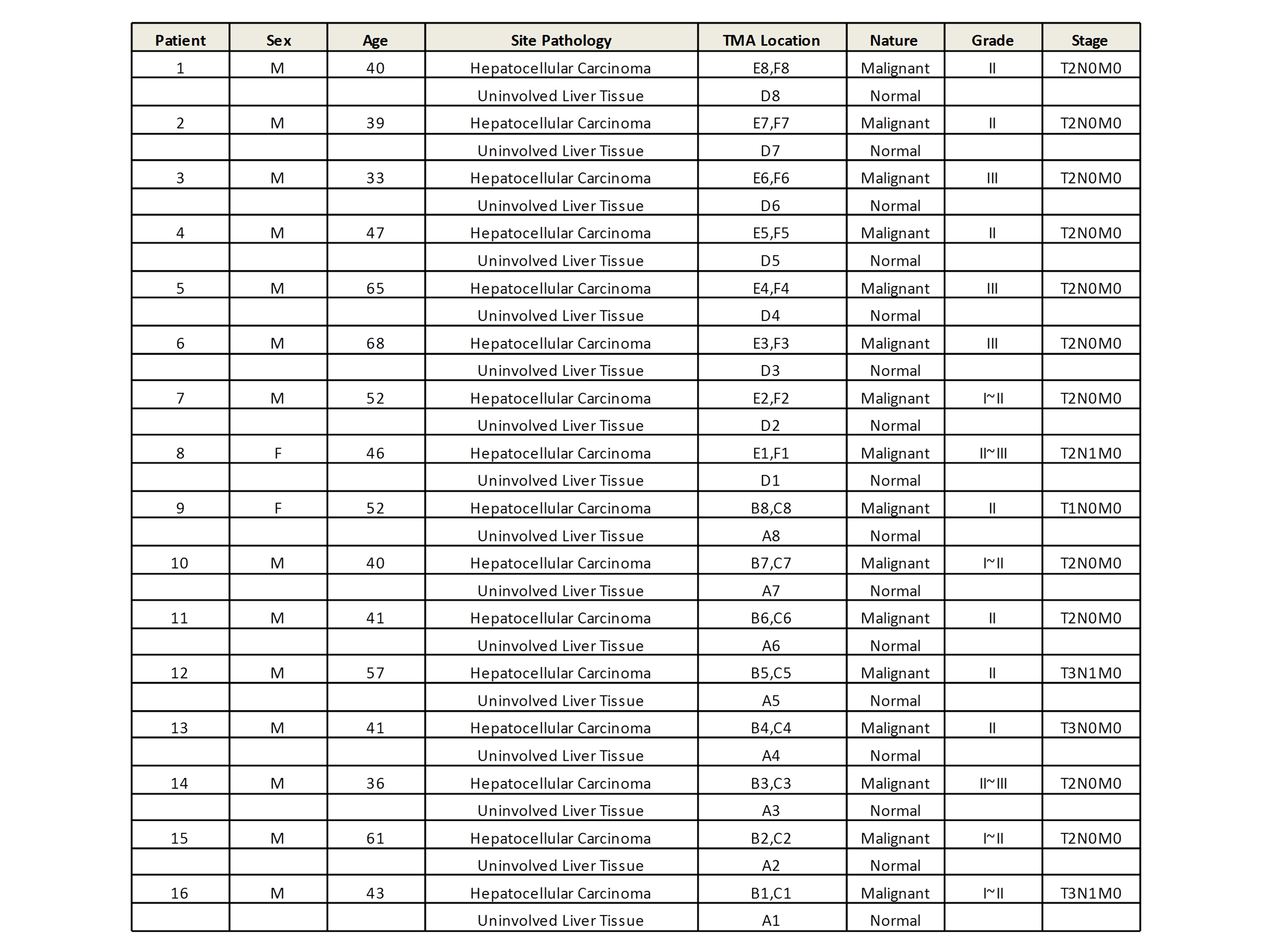

Supplement: Table S1 — Patient Data Summary for Hepatocellular Carcinoma TMA from Biochain. (TIF) [file pone.0106255.s008.tif]
